# Biodegradable Bisvinyl Sulfonemethyl-crosslinked Gelatin Conduit Promotes Regeneration after Peripheral Nerve Injury in Adult Rats

**DOI:** 10.1038/s41598-017-17792-2

**Published:** 2017-12-13

**Authors:** Chien-Hsin Ko, Ming-You Shie, Jia-Horng Lin, Yi-Wen Chen, Chun-Hsu Yao, Yueh-Sheng Chen

**Affiliations:** 1 Graduate Institute of Basic Medical Sciences, China Medical University, Taichung, Taiwan; 2 Department of Traditional Chinese Medicine, Tzu Chi Hospital, Hualien, Taiwan; 3 School of Dentistry, China Medical University, Taichung, Taiwan; 4 3D Printing Medical Research Center, China Medical University Hospital, China Medical University, Taichung, Taiwan; 5 Department of Fiber and Composite Materials, Feng Chia University, Taichung, Taiwan; 6 Graduate Institute of Biomedical Sciences, China Medical University, Taichung, Taiwan; 7 3D Printing Research Center, Asia University, Taichung, Taiwan; 8 Biomaterials Translational Research Center, China Medical University Hospital, Taichung, Taiwan; 9 Lab of Biomaterials, School of Chinese Medicine, China Medical University, Taichung, Taiwan; 10 Department of Bioinformatics and Medical Engineering, Asia University, Taichung, Taiwan

## Abstract

In our previous study, we found that gelatin-based materials exhibit good conductivity and are non-cytotoxic. In this study, gelatin was cross-linked with bisvinyl sulfonemethyl (BVSM) to fabricate a biodegradable conduit for peripheral nerve repair. First, BVSM on the prepared conduit was characterized to determine its mechanical properties and contact angle. The maximum tensile strength and water contact angle of the gelatin-BVSM conduits were 23 ± 4.8 MPa and 74.7 ± 9°, which provided sufficient mechanical strength to resist muscular contraction; additionally, the surface was hydrophilic. Cytotoxicity and apoptosis assays using Schwann cells demonstrated that the gelatin-BVSM conduits are non-cytotoxic. Next, we examined the neuronal electrophysiology, animal behavior, neuronal connectivity, macrophage infiltration, calcitonin gene-related peptide localization and expression, as well as the expression levels of nerve regeneration-related proteins. The number of fluorogold-labelled cells and histological analysis of the gelatin-BVSM nerve conduits was similar to that observed with the clinical use of silicone rubber conduits after 8 weeks of repair. Therefore, our results demonstrate that gelatin-BVSM conduits are promising substrates for application as bioengineered grafts for nerve tissue regeneration.

## Introduction

The nervous system is the fundamental network that controls and regulates bodily functions, which include autonomic regulation, sensations, and movement^1^. Therefore, patients with nerve damage may experience a dramatic decline in their quality of life and severe neurological dysfunction. There are two components to the nervous system, namely the central nervous system which consists of the brain and spinal cord, and the peripheral nervous system which consists of the network of motor and sensory nerves outside of the brain and spinal cord. Damage to the peripheral nervous system (PNS) leads to permanent dysfunction of target tissue, neuropathic pain, and reduced quality of life^2^. Therefore, regenerating damaged PNS is a difficult and challenging problem. For PNS damage less than 5 mm in length, axonal regeneration can occur spontaneously. However, the repaired nerve always loses some function, particularly when the nerve defect or gap is too long. Autologous nerve grafts harvested from functionally less critical areas such as sural nerves and superficial cutaneous nerves are traditionally used to bridge damaged PNS tissue^3^. Therefore, autologous nerve grafts or nerve allografts remain as the current primary treatment method for treating severe traumatic nerve injury with large nerve gaps. Nerve grafts are beneficial for axon recovery because they not only protect, but also guide axon regeneration towards its distal end^4^. They also contain cellular materials that expedite the recovery process. Autologous nerve grafts are extracted from the patient’s own body, and by far are the most effective clinical treatment for peripheral nerve trauma^5^.

However, the availability of autologous nerve grafts is limited, and surgical harvesting of graft tissue may also cause secondary damage or loss of function at the donor site. In situations where autologous nerve grafting is not feasible, allogenic nerve grafts can be applied. Allograft nerve grafts are not extracted from the patient’s body, but rather from another donor or cadaver^6^. These allogenic nerve grafts, however, pose immunogenic problems related to antigenicity and increased morbidity associated with immunosuppression^7^. Thus, researchers have developed acellular human nerve allografts that have been processed by removing immunoreactive components from allogenic nerve grafts while retaining the extracellular matrix components such as growth factors, laminin, and collagen^8^. Only in recent years have methods of treatment for peripheral nerve injury shown better results. The combination of an acellular nerve graft and biocompatible nerve conduits can stimulate profound peripheral nerve recovery^9^. Acellular nerve graft conduits not only prevent chronic foreign body responses, but also are capable of potentially treating lengthy nervous system injuries^10^. Because the diversity of materials for use as nerve conduits has expanded immensely, we can now construct and incorporate optimal materials and support cells depending on the type of nerve conduit required^11^. There are currently numerous commercially available conduits being examined in clinical trials. Researchers have also developed and investigated a wide variety of nerve conduits for enhancing neurogenesis, neuronal differentiation, and angiogenesis for widespread clinical applications.

Nerve conduits provide a reliable structural support that mimics the neural pathway. An ideal nerve conduit should be biocompatible, supportive, flexible, and, most importantly, adequately permeable for the diffusion of oxygen, metabolites, fluids, growth factors, and proteins from the surrounding tissues^12^. Currently, nerve conduits consist of three main types: autogenous or non-autogenous biological, non-degradable (e.g. silicone, elastomer hydrogel, porous stainless steel), and biodegradable (e.g. gelatin, poly(glycolic acid), poly(lactic acid), polyesters, chitosan)^13^. Although many natural and synthetic polymers have been developed, nerve conduits do not match the functional recovery of autografts^14,15^. Therefore, biodegradable nerve conduits are favorable because they induce a limited foreign body response. Some commercially available biodegradable nerve conduits are currently undergoing clinical trials^16^.

In this study, we developed a bisvinyl sulfonemethyl (BVSM)-crosslinked gelatin conduit for peripheral nerve repair which is based on our previous studies. First, we evaluated the mechanical properties, water uptake ratio, and hydrophilicity of the nerve conduits. Biocompatibility analysis and terminal deoxynucleotidyl transferase dUTP nick end labeling (TUNEL) of the gelatin-BVSM conduits were evaluated with Schwann cells. In contrast to clinically used silicone rubber conduits, in this study, we used retrograde labeling of dorsal root ganglia (DRG) in the BVSM-crosslinked gelatin conduits to assess neuronal connectivity. Nuclear factor-κB (NF-κB)-dependent luminescent signal in transgenic mice carrying a luciferase gene accompanied by histochemical assessment was used to assess host-conduit interactions. In addition, we examined calcitonin gene-related peptide (CGRP) in the lumbar spinal cord by immunohistochemistry, and measured morphometric and electrophysiological data in a Sprague-Dawley (SD) rat sciatic nerve defect model. Finally, we observed the macrophage infiltration by immunostaining for Iba1 and CD68 and measured the expression levels of nerve regeneration-related proteins in the regenerated nerves to determine whether the BVSM-crosslinked gelatin conduits could support axonal regeneration and functional restoration.

## Materials and Methods

### Fabrication of Gelatin-BVSM Conduits

A 10% gelatin solution was prepared by stirring gelatin (Sigma #G2500, Saint Louis, MO, USA) into 0.2 M Na_2_HPO_4_ at 60 °C. A mandrel tube composed of silicone rubber (1.96 mm OD; Helix Medical, Inc., Carpinteria, CA, USA) was used to dip into 10% gelatin solution at a constant rate followed by 30 s of soaking. The above coating procedure was conducted a total of 9 times to produce a conduit coated with approximately 400-µm-thick gelatin. The conduit was then air-dried for 1 h followed by 24 h of soaking in 0.3% BVSM (Tokyo Chemical Industry, Tokyo, Japan). After cross-linking, the gelatin-BVSM conduits were washed three times with 95% ethanol and air-dried for 7 days. The conduits were then removed from the mandrel support and cut into 15-mm pieces. Tiny holes were bored into both ends of the conduits for better nerve-conduits adhesion. Next, 25 kGy γ-rays were used to sterilize the specimens prior to cell and animal studies.

### Macroscopic Structure of Gelatin-BVSM Conduits

Scanning electron microscopy (SEM) was conducted to observe the morphology of the gelatin-BVSM explants. A Hitachi E-1010 ion sputter (Tokyo, Japan) was used to coat the specimens with gold and a Hitachi S3000N SEM was used to obtain micrographs at a 5-kV accelerating voltage.

### Mechanical Properties of Gelatin-BVSM Conduits

Mechanical properties were evaluated in dry environments. All specimens were preconditioned in a humidifier with 50% humidity at 23 °C for 2 days. Testing machines from AG-IS, Shimadzu Co. (Kyoto, Japan) were used to analyze the maximum tensile force. All test specimens were cut into the shape of a dumbbell and stretched at a rate of 0.6 mm/min. Five tests were conducted for each specimen and the average was recorded.

### Analysis of Water Contact Angle for Gelatin-BVSM Conduits

First, 20 μL deionized water was pipetted onto the gelatin-BVSM films for contact angle measurement. An autopipette was used to ensure similar volumes of deionized water for each sample. Photos of the water droplet were acquired with the camera after 30 s and contact angles were measured by analyzing the photos with ImageJ software (National Institutes of Health, Bethesda, MD, USA).

### Water Uptake Ratio of Gelatin-BVSM Conduit

The water uptake ratio was measured using weight equilibrium and analyzed using the equation below:

$${\rm{Water}}\,{\rm{uptake}}\,{\rm{ratio}}\,=({\rm{Wt}}-{\rm{W0}})/\mathrm{W0},$$

where Wt is the weight of the test specimens after soaking (swollen test specimens) and W0 is the weight of the test specimens after drying (dried test specimens). The ratio for each procedure was determined at different time-points. Prior to the analysis, the gelatin-BVSM conduit was immersed in 10 mL deionized water at room temperature.

### Biocompatibility and Apoptosis of Gelatin-BVSM Conduits Extracts

Indirect cytotoxicity was determined as described in the modified version of ISO10993-12. Briefly, 6-cm^2^ specimens of the gelatin-BVSM conduit were cleaned several times with phosphate-buffered saline (PBS) and then dried at room temperature with laminar flow. Gelatin-BVSM conduits were soaked in 1 mL of Dulbecco’s modified Eagle’s Medium (DMEM) and incubated with 75% humidity and 5% CO_2_ for 24 h to obtain the gelatin-BVSM extract solution. Simultaneously, RSC96 Schwann cells (10^4^ cells/well) were cultured in a 96-well plate at 37 °C for 24 h, and then the DMEM was removed and changed with gelatin-BVSM extract solution at 200 µL/well. After incubation for 24 and 48 h, the extract solution was removed and 110 µL MTT solution was added to each well (concentration: 5 mg/mL), followed by incubation at 37 °C in an incubator for 4 h. The MTT solution was replaced with 50 mL of dimethyl sulfoxide to dissolve the formazan. A microplate reader (Bio-Tek Instrument, Inc., Winooski, VT, USA) was used to measure color intensity at 550 nm. The data is presented individually for each experiment and as a percentage of the control level of the optical density. Cellular toxicity was also determined in this assay. After 48 h treatment with gelatin-BVSM extracts, the cells were rinsed with PBS three times, followed by fixation for 30 min with 2% paraformaldehyde, and then permeabilized with 0,1% Triton X-100 with PBS at room temperature for 30 min. After washing with PBS, the TUNEL assay was then performed according to the manufacturer’s instructions. Specimens from all groups were immersed in TUNEL buffer and incubated at 37 °C in a humidified chamber in the dark for 1 h, washed twice with PBS, and immersed in 40,6-diamidino-2-phenylindole (DAPI, Sigma, 1 mg/mL) at 37 °C for 20 min. Positive control for the experiment was 4% dimethyl sulfoxide (DMSO) in DMEM medium. All stained were considered as cells that had undergone apoptosis and were detected with a fluorescence microscope (DP70, Olympus, Tokyo, Japan).

### Biocompatibility of Gelatin-BVSM Conduits

All *in vivo* experimental protocols were conducted according to standard guidelines and regulations and were approved by the Ethical Committee for Animal Experiments of China Medical University, Taichung, Taiwan. NF-κB-responsive elements promoter for luciferase genes were produced as described in our previous studies and inserted into transgenic mice^17,18^. The transgenic mice were bred with wild-type F1 mice to give rise to NF-κB-luc heterozygous mice harboring an FVB genetic background. Anesthesia with 0.12 g ketamine/kg body weight prior to insertion of the gelatin-BVSM implantation was performed in the transgenic mice. A 3-mm incision was made on the mice back for implantation. The gelatin-BVSM conduit was placed subcutaneously and the incision was closed with silk sutures. For this study, six mice were randomly selected and placed into two groups of three mice each: (i) sham group, mice with nil implantations and (ii) gelatin-BVSM group, mice with gelatin-BVSM conduit implantations. Luciferase activity was imaged at 1, 3, 7, and 28 days, followed by histochemical staining. Anesthesia with isoflurane and 150 mg/kg body weight of luciferin were injected intraperitoneally prior to *in vivo* imaging. After 5 min, the IVIS Imaging System 200 Series (Xenogen, Hopkinton, MA, USA) was used to image the mice. The mice were laid prone in the imaging chamber and imaged for 5 min with the highest sensitivity setting. Living Image software (Xenogen) was used to quantify the photons emitted from tissues. Signal intensity was calculated as the sum of all detected photons within the area of interest for each second, excluding background luminescence (photon counts).

For histochemical staining, the mice were sacrificed and gelatin-BVSM conduits were removed and treated with 10% formalin for 48 h. Tissues were rinsed with PBS and dehydrated using a series of diluted and graded alcohols of 50%, 70%, and 95%. Tissues were soaked in each concentration of ethanol for 30 min. All specimens were fixed in paraffin before cutting into 12-mm slices. Hematoxylin and eosin staining was used for histomorphometric evaluations. For tissues reactivity evaluation, optical microscopy was used to observe the consistency and size of foreign body capsules and inflammatory responses.

### Gelatin-BVSM Conduits Implantation

*In vivo* studies were performed under general anesthesia on 30 adult male SD rats classified into 3 time groups of 2, 5, and 8 weeks with 10 rats in each group subjected to implantation of gelatin-BVSM conduits. Anesthesia using the inhalational technique (AErrane, Baxter, Deerfield, IL, USA) was conducted prior to this test. After skin incision, muscles and fascia tissues were separated by blunt dissection, followed by transection of the right sciatic nerves into two segments, namely the distal and proximal segments. Both segments were secured with a single 9–0 nylon suture through the epineurium with the outer lumen of the conduits. The segments were attached at a depth of 2.5 mm, leaving a gap of 10 mm. 4–0 chromic gut sutures were used to re-approximate the layer of muscle, and the incision was closed using 2–0 silk sutures. The animals were housed in humidified rooms at 22 °C and 45% humidity with 12-h light cycles. Food and water were provided *ad libitum*. Clinically approved silicone rubber conduits were used as the control group.

### Electrophysiological Analysis

All of the animals were anesthetized again and their sciatic nerves were exposed for electrophysiological testing. Both the stimulating cathode and anode were placed at the following locations: the sciatic nerve trunk 5 mm proximal to the transection site with the anode placed 3 mm proximally to the cathode, respectively. Both probes were composed of a stainless-steel monopolar needle. A computer system from Biopac Systems, Inc. (Goleta, CA, USA) was used to measure and record the amplitude, latency, and nerve conductive velocity (NCV) from the evoked muscle action potentials (MAPs) of the gastrocnemius muscles. Micro-needle electrodes were used to detect the MAPs. A stimulus to launch the first negative deflection was considered as latency. The areas under the curves (measured from baseline to the maximal negative peak) and amplitude were also determined. Finally, the NCV was carried out by placing the recording electrodes in the gastrocnemius muscles and stimulating the sciatic nerve proximally and distally to the bridging conduit. The NCV was then obtained by dividing the distance between the stimulating sites by the difference in latency time.

### Retrograde Labeling with Fluorogold

The method used for fluorogold-labeling of neurons has been described previously^19,20^. Briefly, a 2% fluorogold suspension was prepared by dissolving fluorogold in distilled water. This solution was stored at 4 °C in the dark. The fluorogold solution was directly injected with a Hamilton micro-syringe into the common peroneal nerve and posterior tibia nerve after electrophysiological recording. After fluorogold injection for 5 days, the rats were transcranially perfused sequentially with 200 mL of 0.9% saline, followed by cold 4% paraformaldehyde in 0.1 M PBS. Next, L4 and L5 DRGs on the same side of the injury were dissected and removed and then soaked overnight in 4% paraformaldehyde for post-fixation before additional overnight soaking in 30% phosphate-buffered sucrose solution. A cryostat was used to freeze longitudinal sections of the spinal cord and obtain DRGs of 40 μm thickness. After 30 min of drying, the section was mounted and analyzed under an ultraviolet fluorescence microscope (Olympus Ckx41).

### Histological Processing

For histological processing, the animals were perfused transcranially similar to as described above. The L4 spinal cord was then detached and post-fixed in the same fixative material for 4 h. For cryo-protection, all specimens were immersed in 30% sucrose at 48 °C overnight and then embedded in the cutting temperature solution. The sample was frozen at −20 °C, followed by slicing into 18-mm slices with a cryostat. The sliced samples were fixed on poly-L-lysine coated slides. Immunohistochemistry analysis was carried out in a two-step protocol in accordance with the manufacturer’s instructions (Novolink Polymer Detection System, Novocastra, Buffalo Grove, IL, USA). The specimens were incubated in 0.3% H_2_O_2_ to inactivate all endogenous peroxidase activity, and then immersed in Protein Block (RE7102; Novocastra) solution to inhibit all non-specific binding sites. All specimens were treated serially with anti-CGRP antibody 1:1000 (Calbiochem, San Diego, CA, USA), Post Primary Block (RE7111; Novocastra), and secondary antibody (Novolink Polymer RE7112), after which diaminobenzidine solution was used to develop the L4 spinal cord sections and hematoxylin was used for counterstaining. All steps were conducted under a microscope. In the chamber, sciatic nerve sections were removed from the middle regions of the regenerated nerve and fixed, and then treated with 0.5% osmium tetroxide before being dehydration and embedding in Spurr’s resin. Next, 2-μm-thick sections were cut using Leica EM UC6 microtome (Leica Biosystems, Wetzlar, Germany) diamond knife before staining with toluidine blue and observation under an optical microscope (Olympus IX70; Olympus). In addition, 70-nm ultra-thin sections were cut and stained with uranyl acetate and lead citrate before transmission electron microscopy (TEM) analysis at 100 kV (Leica).

For immunofluorescent staining of macrophages, specimens were treated with 10% bovine serum albumin containing 0.4% Triton X-100 for 1 h, followed by 4 °C overnight treatment with primary antibodies in blocking solution. The primary antibodies were anti-CD68 (1:200; Serotec, Hercules, CA, USA), and anti-Iba1 (1:100, Bioss, Woburn, MA, USA). Tissue sections were rinsed three times with PBS and immersed in appropriate secondary antibodies tagged with Alexa Fluor 488 or 594 (1:500; Abcam, Cambridge, UK) in the dark for 1 h at room temperature. Finally, the coverslip was mounted onto the slide with aqueous-mount mounting medium (ScyTek, Logan, UT, USA) and images were acquired with an SP2/SP8X confocal microscope (Leica).

### Image Analysis

The image analyzer system (Image-Pro Lite, Media Cybernetics, Rockville, MD, USA) coupled to a microscope was used to observe all tissue specimens. CGRP-immunoreactivity in the dorsal horn of the lumbar spinal cord was detected by immunohistochemistry analysis as described previously^21^. Immunoproducts were considered positive if they were 5-fold denser compared to the background levels. A 400x magnification was used to observe the ratio of areas filled with positive CGRP-immunoreactive cells in the dorsal horn ipsilateral to the injury following neurorrhaphy relative to the lumbar spinal cord. The quantity of neural components in each section was also measured. For further observations and measurements of myelinated axons, at least 50% of the sciatic nerve section was randomly chosen from each specimen. An area algorithm was used to determine the total number of axons for each nerve. Total nerve areas were evaluated under a microscope at 40x. In addition, macrophage density was measured by dividing macrophage counts by total nerve areas. Finally, blood vessels in the endoneurium were counted at 400x.

### ELISA Analysis

Production of insulin-like growth factor (IGF)-1, brain-derived neurotrophic factor (BDNF), and glial cell line-derived neurotrophic factor (GDNF) was quantified using ELISA kits (Abcam) in accordance with the manufacturer’s instructions. Briefly, total blood (10 mL) was collected from the exposed heart at the time of decapitation of deeply anesthetized animals and incubated at room temperature for 30 min. Next, the serum was obtained by centrifuging the total blood at 2000 rpm at 4 °C for 15 min and analyzed using an ELISA kit. The concentrations of IGF-1, BDNF, and GDNF were measured by correlation with a standard curve. Blank disks were analyzed as a control.

### Statistical Analyses

For statistical analysis of *in vivo* experiments, data were collected by the same observer and expressed as the mean ± standard deviation (SD). Raw data for the histological and electrophysiological analyses are shown in Table 1. Groups were compared by t-test of variance using SAS 9.4 (SAS Institute, Inc., Cary, NC, USA). Statistical significance was set at P < 0.05.

**Table 1:** Raw data for the histological and electrophysiological analyses.

Group	Total nerve area (mm^2^)	No. of axons (#)	NCV (m/s)	Latency (ms)	Amplitude (mV)	MAP area (mVms)
gelatin-BVSM 5 week	0.03	122	28.33	1.36	9.54	8.83
	N/A	N/A	27.23	1.39	9.45	12.35
	0.18	1934	27.40	1.35	4.67	7.06
	0.12	886	28.93	1.31	7.54	8.23
	0.11	571	35.23	1.08	10.95	14.05
	0.07	515	31.83	1.13	6.17	8.73
	0.11	857	33.05	1.09	9.42	11.37
	0.07	943	30.33	1.25	7.92	9.83
	0.09	672	28.73	1.32	6.58	7.92
	0.06	476	34.50	1.10	6.67	11.87
Mean	0.09	775	30.55	1.24	7.89	10.02
SD	0.04	503	2.78	0.12	1.83	2.15
gelatin-BVSM 8 week	0.41	3154	31.00	1.22	5.25	7.68
	0.22	1907	31.90	1.19	9.29	10.20
	0.41	5465	27.68	1.38	12.54	14.30
	0.22	331	33.30	1.13	8.17	9.62
	0.04	558	35.30	1.08	11.40	8.12
	0.31	4215	33.93	1.06	16.18	16.65
	N/A	N/A	34.85	1.09	10.53	15.35
	0.26	2667	35.48	1.07	5.58	9.58
	1.25	12315	35.33	1.08	9.80	15.35
	0.04	163	32.88	1.15	12.68	15.55
Mean	0.35	3419	33.16	1.14	10.14	12.24
SD	0.36	3795	2.46	0.10	3.33	3.49
Silicone 8 weeks	0.28	1514	28.93	1.31	6.29	9.42
	0.21	3227	27.70	1.37	3.75	4.50
	0.36	4585	30.35	1.25	5.42	9.09
	0.31	5213	28.90	1.31	11.68	13.93
	N/A	N/A	33.33	1.14	7.04	9.09
	0.21	3745	29.45	1.29	7.17	7.37
	N/A	N/A	31.78	1.20	10.25	12.42
	N/A	N/A	34.50	1.10	10.50	10.37
	N/A	N/A	28.63	1.33	11.00	11.58
	0.28	3105	33.18	1.14	10.68	12.30
Mean	0.28	3565	30.67	1.24	8.38	10.01
SD	0.06	1290	2.36	0.09	2.77	2.75

## Results

### Characterization of Gelatin-BVSM Conduits

**Figure 1:**
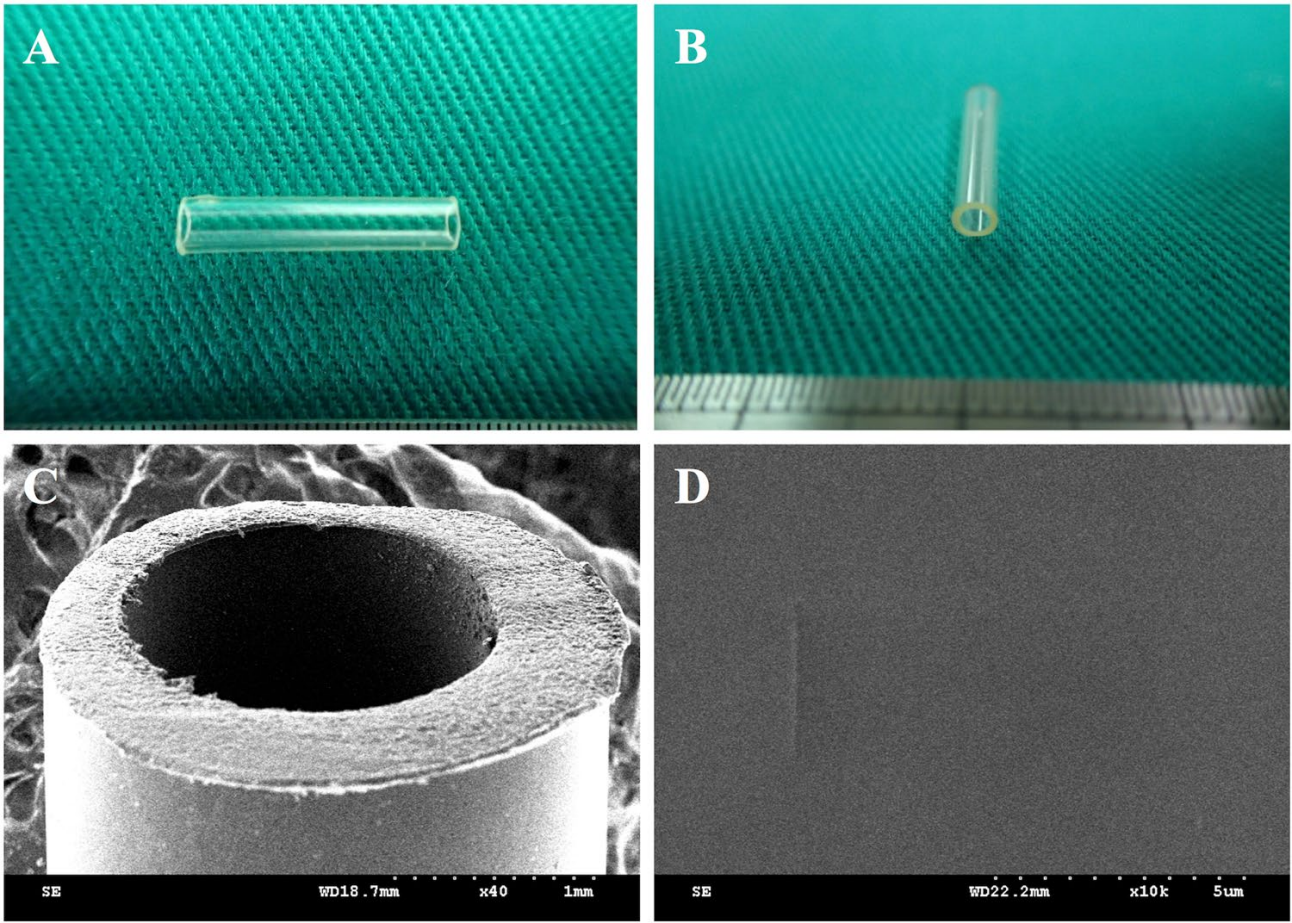
(**A**,**B**) Overview, optical micrographs, and SEM micrograph with (**C**) low and (**D**) high magnification of BVSM-gelatin conduits.

The gelatin-BVSM nerve conduits were transparent and concentric with both smooth inner and outer lumen surfaces (Fig. 1). The gelatin-BVSM conduits had a cross-linking index of 58 ± 4%, expressed as the percentage of free amino groups lost during cross-linking. The maximum tensile strength of the conduits was 23 ± 4.8 MPa with a water contact angle of 74.7 ± 9°. The gelatin-BVSM conduits were soaked for 84 h and water uptake ratios were analyzed at different time points during the 84 h. For the first 6 h, there was a substantial increase in conduit weight, followed by a reduction in water uptake which led to a plateau after 12 h of soaking. The lumens and integrity of external wall remained intact, even after 84 h of soaking, with a weight increase of 235%.

### Cytotoxicity and Apoptosis of Gelatin-BVSM Conduits

**Figure 2:**
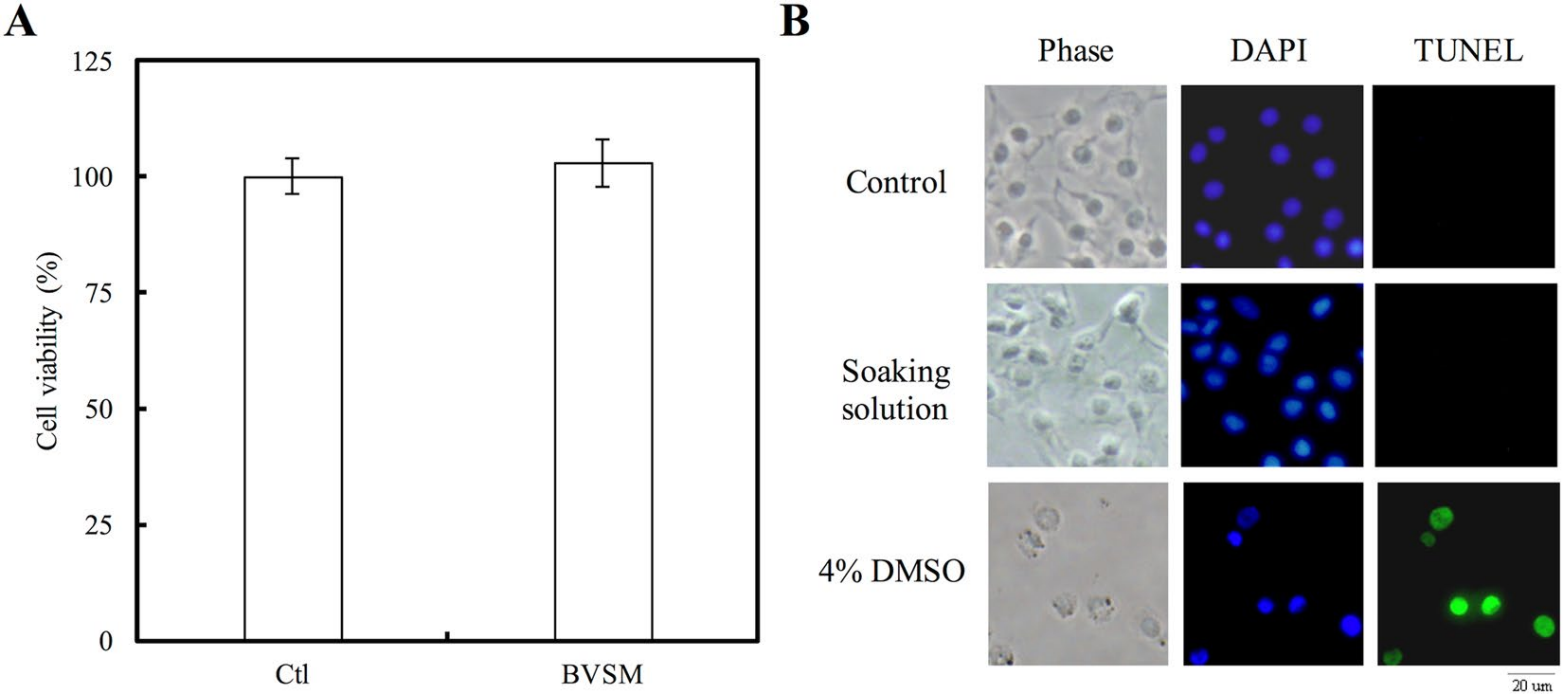
Induction of biocompatibility using extract solution of BVSM-gelatin conduits. (**A**) Quantification of cytotoxic test of extract solutions of BVSM-gelatin conduits relative to controls on Schwann cells. (**B**) Nuclei of Schwann cells were characterized by DAPI and TUNEL assay and investigated under a fluorescence microscope. Scale bars: 50 µm.

The cytotoxicity test showed that gelatin-BVSM nerve conduits were non-toxic to Schwann cells. The results of the MTT assay revealed no significant difference between the cell viability of Ctl and gelatin-BVSM, indicating that the gelatin-BVSM nerve conduits were suitable for cell culture (Fig. 2A). In addition, the extracts from gelatin-BVSM nerve conduits did not induce cell apoptosis. No TUNEL-positive cells were observed in Fig. 2B, therefore suggesting that there was no fragmentation of DNA in the Schwann cells. These results indicated that gelatin-BVSM conduits were not cytotoxic towards cultured cells. It was also confirmed by our imaging probe of the apoptosis TUNEL assay, which showed that 4% DMSO induced significant apoptosis, or more TUNEL-positive immuno-labeling.

### Tissue Reactions to Gelatin-BVSM Conduits

**Figure 3:**
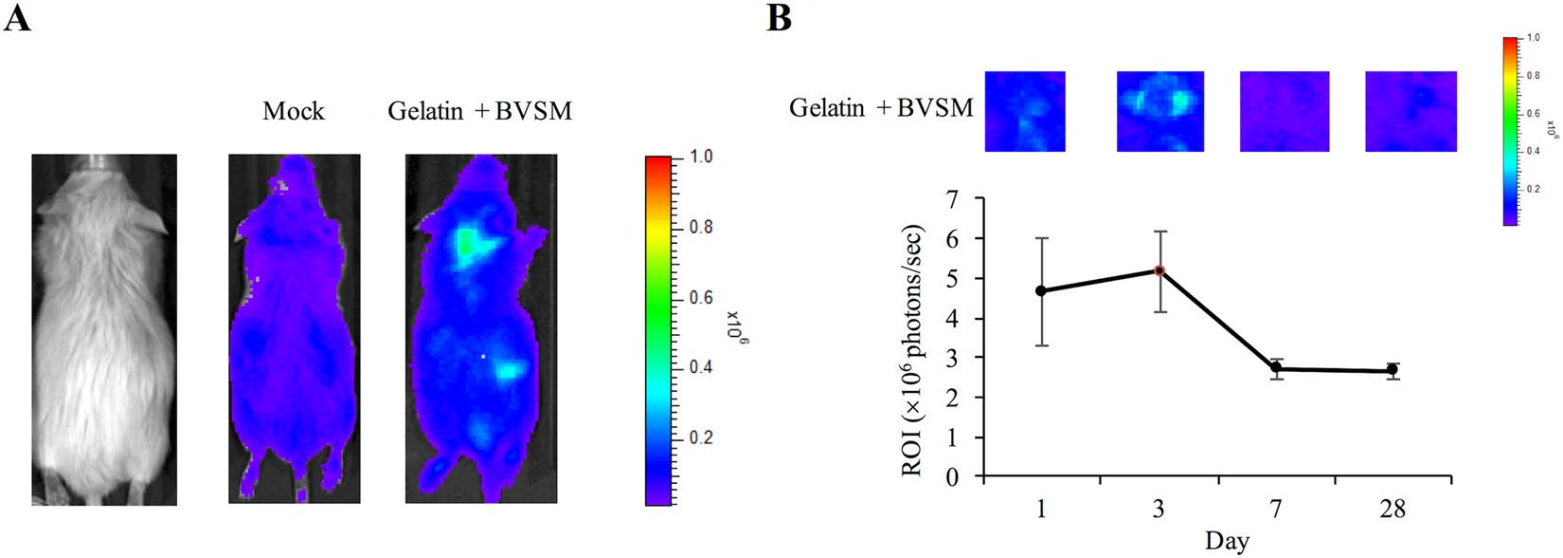
NF-κB-dependent bioluminescence in living mice implanted with BVSM-gelatin conduits. (**A**) Diagrams show the bioluminescent signal within a radius of 2.5 mm of implanted region (boxed area). The color overlay on the image represents the photons s21 emitted from the animal, as indicated by the color scales. (**B**) Quantification of photon emission within the implanted region.

**Figure 4:**
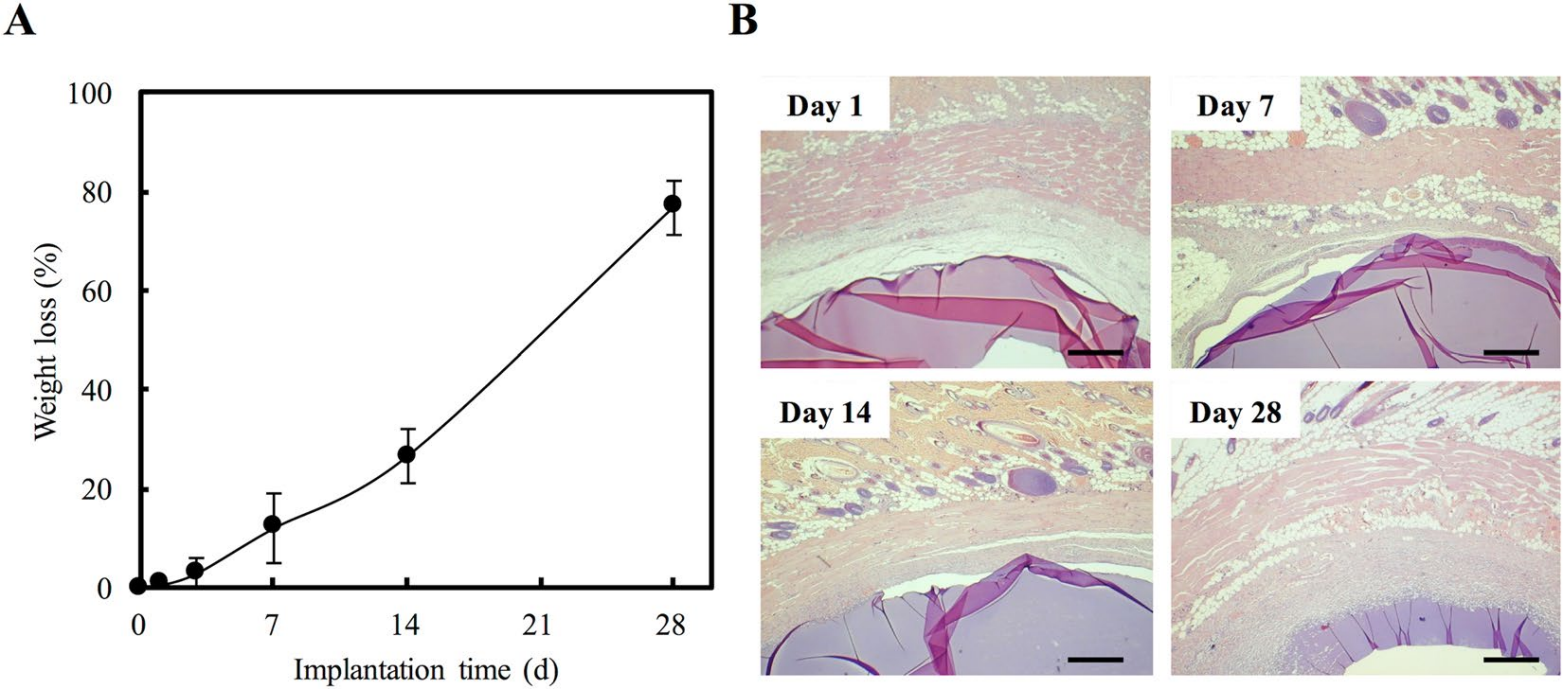
(**A**) Weight loss and (**B**) micrograph of interface area of BVSM-gelatin conduits after implantation for different times. Scale bars: 100 µm.

After the postoperative period, there were no signs of inflammatory responses to foreign bodies or necrotic tissues in any of the rats. The implants were placed subcutaneously on the back of the mice and luminescent imaging was used to detect NF-κB-driven bioluminescent signals at various time points (Fig. 3A). The luminescent signal increased during the initial stages and then dramatically decreased (Fig. 3B). NF-κB activity was optimal at day 3, when strong local *in vivo* bioluminescence was detected surrounding the implantation site. Degradation behavior of the gelatin-BVSM nerve conduits was evaluated at different times points from days 1 to 28. As shown in Fig. 4A, the gelatin-BVSM nerve conduits showed weight loss of 26.6% after 2 weeks. Approximately 76.8% of the weight lost was observed after soaking. Different clinical applications require biomaterials with different degradation rates. Consistent with the results of bioluminescent signal measurement, a post-implantation acute inflammatory response was observed (characterized by rapid aggregation of cells resembling lymphocytes and macrophages) at the point when gelatin-BVSM nerve conduits contacted their surrounding tissues. This was observed at 1 day post-implantation (Fig. 4B). However, the lumens and integrity of the wall were still intact at this time point. At day 7, the entire implant was surrounded by a delicate and fibrous capsule composed of tissues with diffused neocapillaries. Inflammatory responses were still observable based on the presence of abundant inflammatory cells. Phagocytosis was observed after 14 day post-implantation at the interfaces between the conduits and tissues. After 28 days, the fibrous tissue capsules became thicker and denser with active neovascularization. Inflammatory responses were continuous throughout this period and macrophages digested the fragmented gelatin-BVSM materials.

### Electrophysiological Measurements

**Figure 5:**
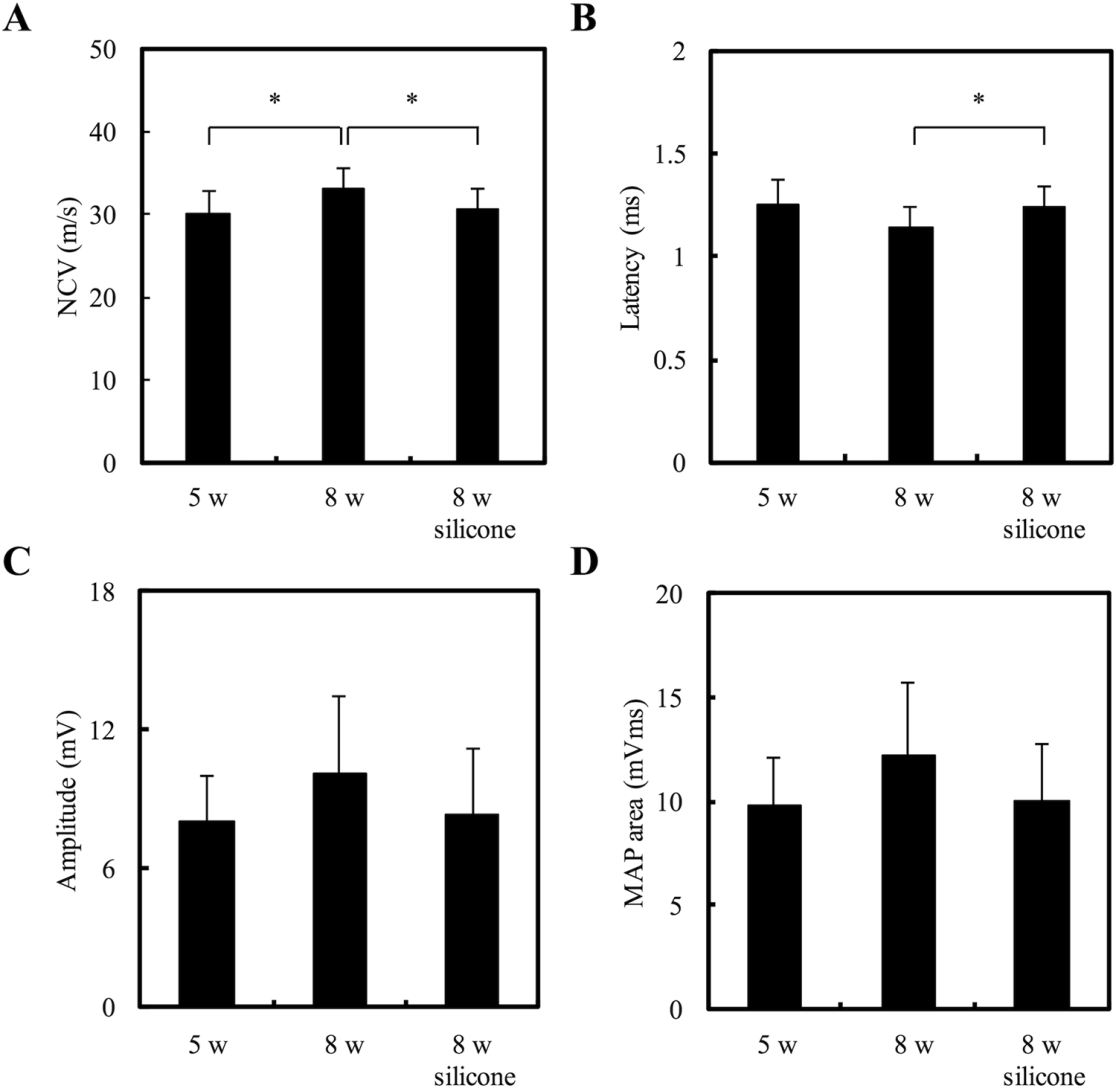
Analysis of evoked muscle action potentials, including (**A**) NCV, (**B**) latency, (**C**) peak amplitude, and (**D**) MAP area. *Indicates a significant difference (P < 0.05) from other examined time points.

For electrophysiological analysis, obvious excitability and conductivity were observed in all rats, indicating that the regenerated nerve fiber had successfully reinnervated with the gastrocnemius muscle. Quantitative data demonstrated that nerve functions, including NCV (Fig. 5A) and latency (Fig. 5B), were improved significantly in the gelatin-BVSM nerve conduits compared to the silicone rubber group after 8 weeks (P < 0.05). The amplitude (Fig. 5C) and MAP area (Fig. 5D) of the regenerated nerves after 8 weeks were higher than in the silicone rubber group, but the differences were not significant (P > 0.05).

### Retrograde Labeling with Fluorogold

**Figure 6:**
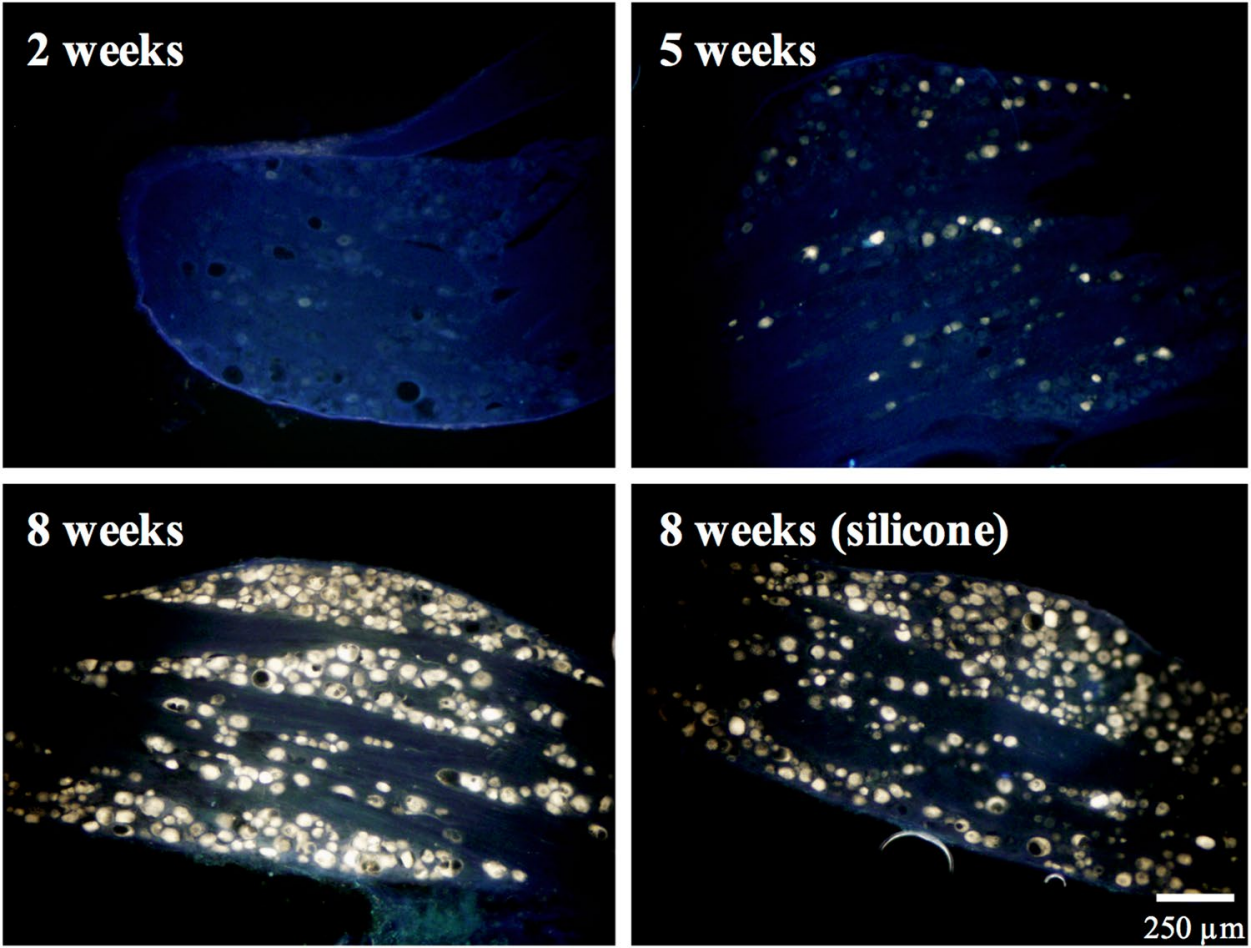
Representative images of the retrograde axonal tracing with fluorogold for different time points. Scale bar: 250 μm.

In the cryostat sections, fluorogold-labeled cells showing migrating axons overcame the bridging nerve tissue and therefore reached the DRG, indicating successful neuronal connectivity (Fig. 6). The density of fluorogold-labeled cells in the DRGs increased significantly in the gelatin-BVSM nerve conduits over the 8 weeks after implantation. In addition, the number of fluorogold-labeled cells in the gelatin-BVSM nerve conduits was similar to clinically used silicone rubber conduits after 8 weeks of repair.

### Effect of Gelatin-BVSM Conduits on Maturity of Regenerated Nerves

**Figure 7:**
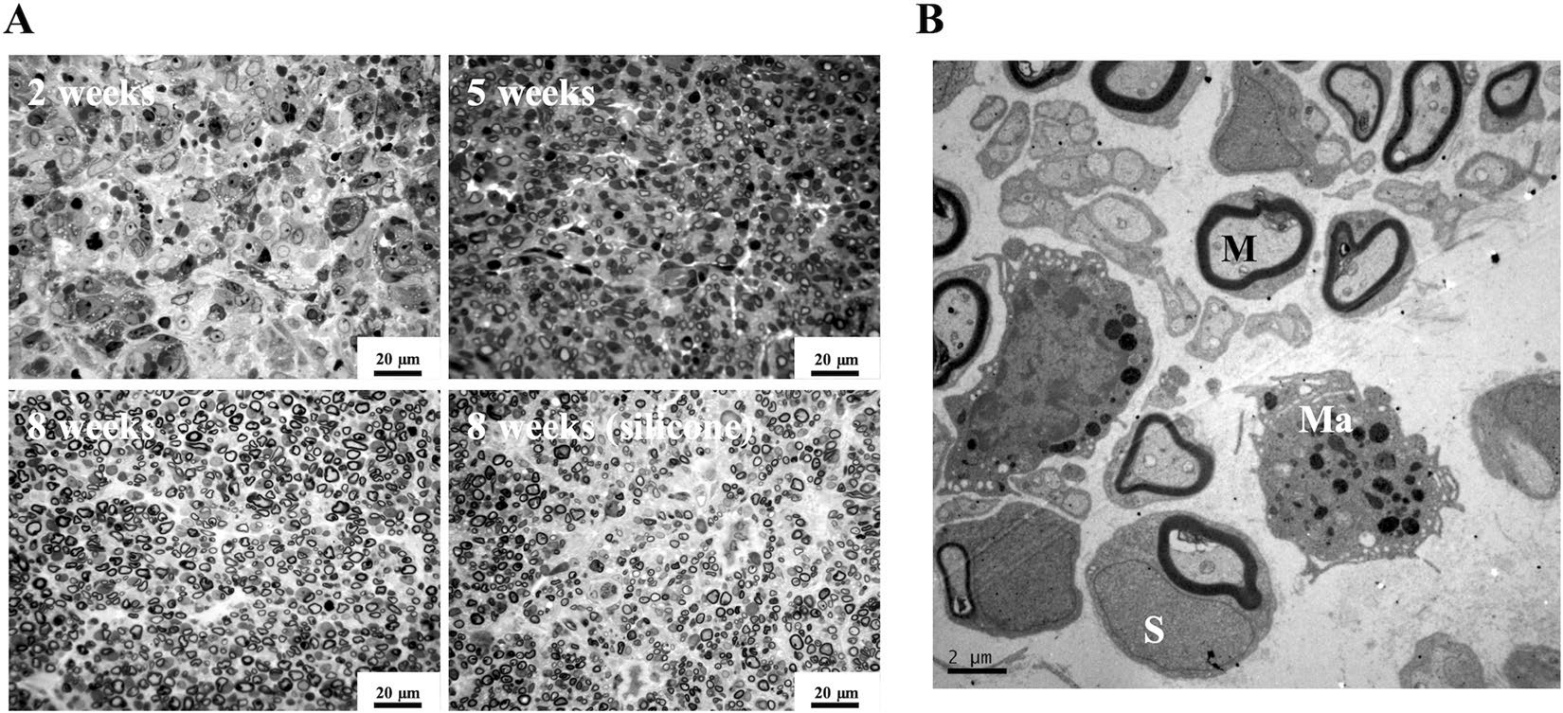
(**A**) Representative micrographs of cross-sections of regenerated sciatic nerves. (**B**) Ultrastructural observation using TEM to show macrophage (Ma), myelinated axon (M), and Schwann cell (S) infiltration and remyelination in regenerated nerves.

The extent of nerve-conduit-supported neuro-regeneration was visually determined as the number of myelinated nerve axons enclosed within the epineurium at random cross-sections of the nerves. At 2 weeks of regeneration, red blood cells and mast cells were present in the regenerated nerve cables, but no myelinated axons were observed (Fig. 7A). At 5-week post-recovery, there was ample vascularization in the regenerated nerves and abundant myelinated axons in the endoneurium. As expected, the quantity of regenerated axons increased progressively throughout the 8-week nerve regeneration process. Additionally, the gelatin-BVSM nerve conduits showed comparable efficiency in supporting nerve regeneration to the silicone-based conduits based on their respective myelinated axonal distribution at 8 weeks after implantation. In addition, an ultrastructural assay of representative regenerated nerves was conducted using TEM. A relatively large fraction of the core in the regenerated cable contained collagenous endoneurial connective tissue and several myelinated axons (Fig. 7B). In addition, endoneurial macrophages were proximity to Schwann cells with myelinated axons, further indicating the roles of macrophages in infiltration and remyelination.

### Morphometric Measurements

**Figure 8:**
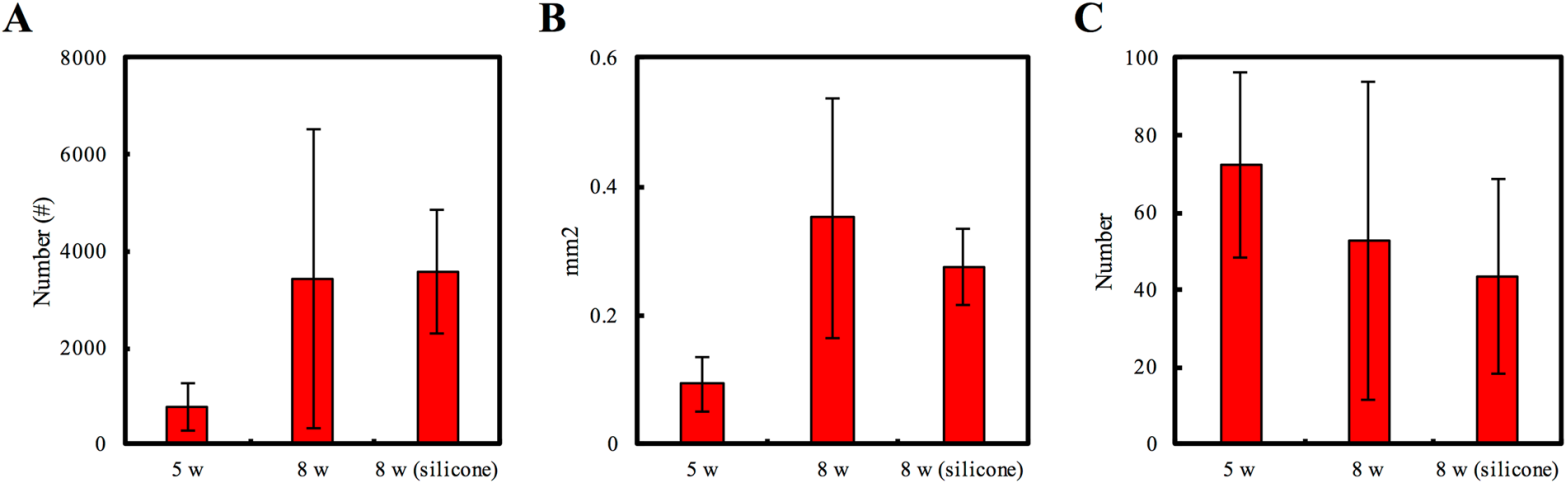
Morphometric analysis of regenerated nerves in the BVSM-gelatin conduits after implantation for 5 and 8 weeks, (**A**) axon number, (**B**) total nerve area and (**C**) blood vessel number.

Morphometric studies showed progress in the recovery stages of regenerated nerves throughout the experimental period, suggesting that the damaged nerve sufficiently regenerated in our gelatin-BVSM nerve conduits. Figure 8A shows the drastic increase in the number of myelinated axons from weeks 5 to 8 of nerve regeneration. Digital analysis showed that the number of axons increased by approximately 4-folds during weeks 5 to 8 of nerve regeneration. Myelinated axon growth supported by the gelatin-based nerve conduit and silicone-based nerve conduit was approximately the same. Figure 8B shows the total cross-sectional area of the regenerating nerve specimen. Furthermore, the gelatin-BVSM nerve conduit supported the regeneration of nerve tissue such that the total surface area of the nerve increased by at- least 3.9-fold from weeks 5 to 8 of nerve regeneration. After 8 weeks of nerve-conduit-supported regeneration, the total nerve area within the gelatin-based conduits was increased by an average of 1.28-fold compared to that with silicone-based control conduits. The blood vessel count in the gelatin-BVSM conduits from weeks 5 to 8 of nerve regeneration was about the same as that observed in the silicone rubber conduit after 8 weeks of implantation (Fig. 8C).

### Macrophages Recruited in Distal Nerve Ends

**Figure 9:**
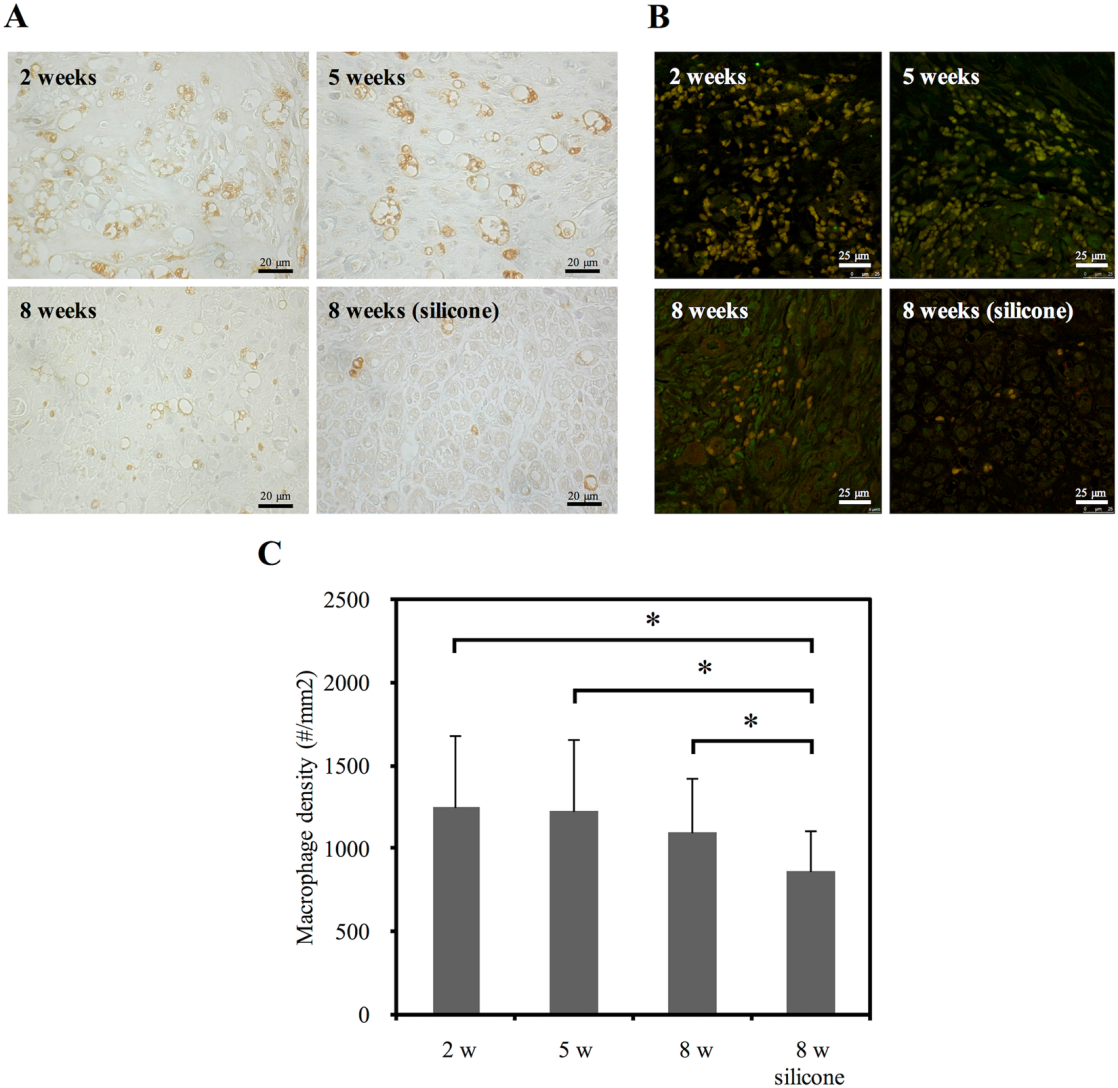
(**A**) Photomicrographs demonstrating anti-rat CD68 immunoreactivity in macrophages from cross-sections of distal nerve cables after BVSM-gelatin or silicone rubber conduits implanted for different times. (**B**) Representative photographs of CD68 and Iba-1 immunoreactivity on the macrophages. (**C**) Quantitation of macrophage infiltration density. *Indicates a significant difference (P < 0.05) from other examined time points.

Immunostaining data for Iba1 and CD68 to quantify macrophages reflected that macrophage density decreased over the implantation period (Fig. 9). A significantly lower density of macrophages in the nerve stumps was observed in the silicone rubber implants compared to in the gelatin-BVSM nerve conduits (P < 0.05). This indicates that the silicone rubber was relatively stable and had inert properties compared to the gelatin-BVSM implants.

### CGRP-IR in the Spinal Cord

**Figure 10:**
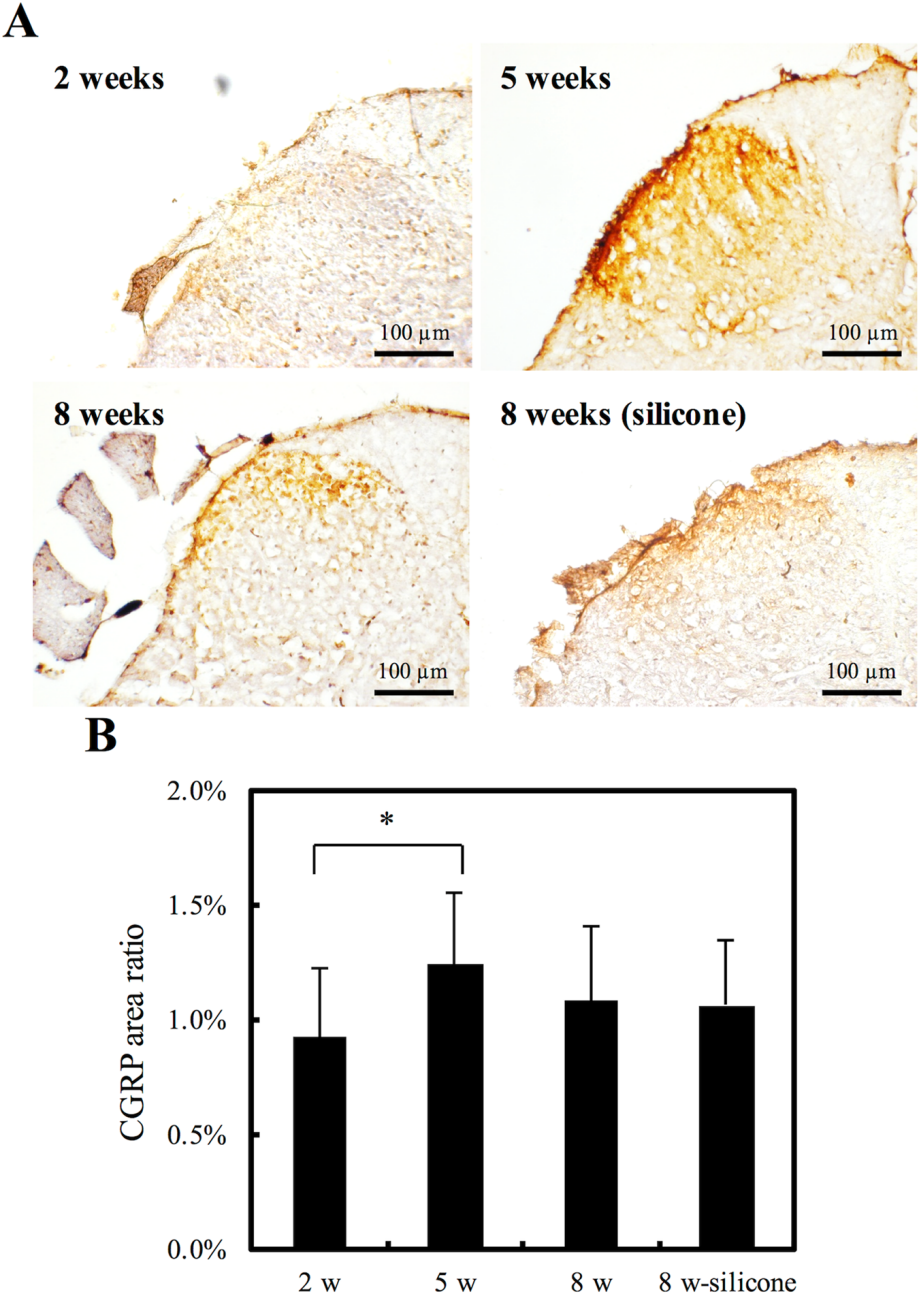
(**A**) Representative histological micrographs of CGRP expression and (**B**) Quantitation of the ratio of CGRP expression area. *Indicates a significant difference (P < 0.05) from other examined time points.

*In vivo* CGRP-immunoreactivity was observed by optical microscopy to examine the extent of neuroregeneration, as CGRP is a nerve-regeneration-promoting peptide. Figure 10 shows that the gelatin-BVSM nerve conduits promoted progressive nerve tissue regeneration, as CGRP expression increased significantly from weeks 2 to 5 (P < 0.05); from weeks 5 to 8, there was a notable decrease in CGRP expression. CGRP expression was approximately the same on the gelatin-based material as on the silicone-based material after 8 weeks of implantation. Thus, dynamic CGRP expression changes occurred in the lumbar spinal cord during the recovery stages of the regenerating nerves while still in the conduits.

### Protein Expression

**Figure 11:**
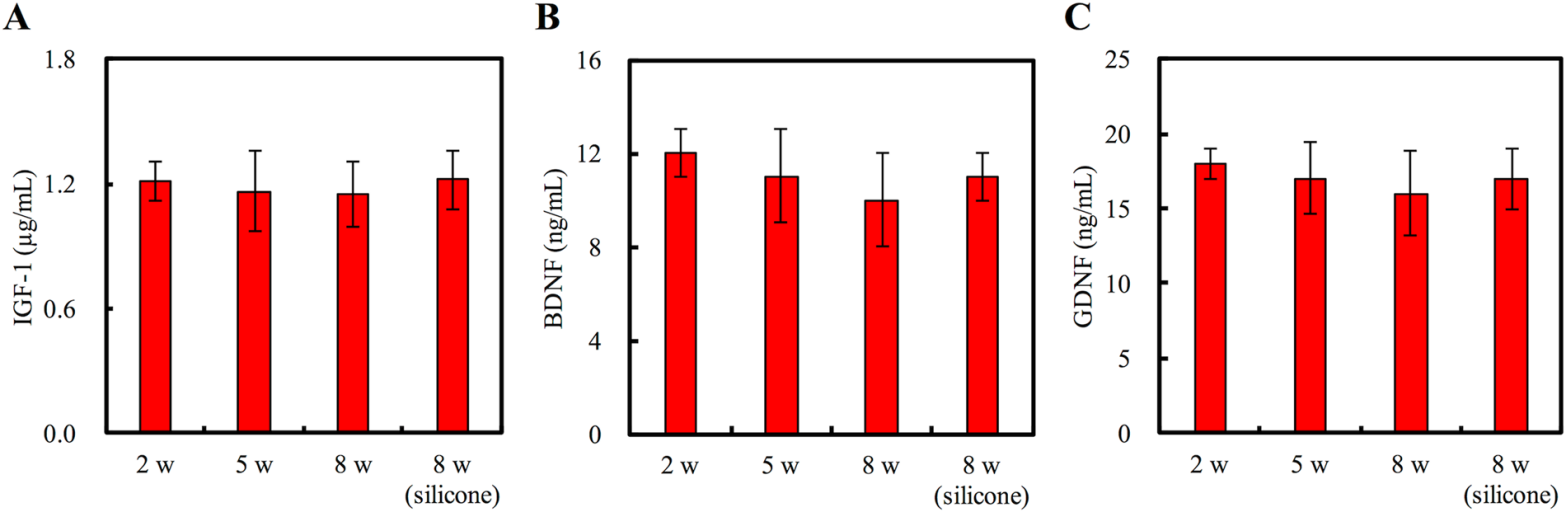
Effects of BVSM-gelatin conduits on proteins expression of (**A**) IGF-1, (**B**) BDNF, and (**C**) GDNF. Values represent the means ± standard deviation (SD) for 10 rats in each group.

The regenerative ability of nerves strongly relies on the regulation of growth factors, which was analyzed using ELISA. Figure 11 shows that the levels of IGF-1, BDNF, and GDNF in the regenerated nerves remained relatively consistent throughout the regeneration process. In addition, the protein expression levels of all three factors in the nerves bridged with gelatin-BVSM nerve conduits after 8 weeks did not differ significantly from those in the silicone rubber conduits. This result indicates that cellular and tissue activity during regeneration within both the nerve guides were similar.

## Discussion

Ideal conduits should be biocompatible, structurally supportive of axonal regeneration and neuronal re-innervation, flexible, and transparent for better visual observation during fixation. The biocompatibility of the conduit should not evoke an immune response after implantation, as an immune response at the site of insertion not only hinders neuron regeneration, but also causes detrimental side effects. Most importantly, the central goal of a conduit is to enable axonal re-innervation of the proximal to distal end of the severed nerve; therefore, it is crucial that conduits ensure a direct pathway for axonal growth. Next, ideal conduits should be constructed with materials that enhance neuron regeneration by both stimulating vascularization and allowing for accumulation of neurotrophic factors. Finally, the success of conduit administration relies heavily on proper placement of the conduit; thus, it is crucial that the conduits have a reasonable level of transparency and thickness. Because neuron repair is extremely difficult, it is imperative that conduits fulfill all requirements and environmental conditions for neuron regeneration. Injuries to nerves, particularly peripheral nerves, are common conditions often diagnosed in clinical settings. In such cases, autologous nerve grafts are the most common technique used for peripheral nerve reconstruction. However, this method has various limitations, such as morbidity at the donor site and the shortage of donated nerves. This led to a search for newer and improved methods; nerve allografts are considered as an alternative, but few successful cases have been reported because of immunological rejections. Therefore, the use of artificial guides for peripheral nerve reconstruction has surfaced recently as an alternative. Over the past few years, numerous clinical and experimental studies have explored possible biomaterials for use as artificial guides. Silicone rubber, collagen^22^, gelatin^23^, chitosan^24^, and poly-ε-caprolactone^25^, are among of various biomaterials considered for the construction of the artificial guides.

A gelatin-based degradable nerve conduit was fabricated and analyzed in this study. It is well-known that gelatin-based hydrogels have a high swelling ability and low mechanical properties^26^. In previous studies, we used genipin-cross-linked gelatin as a bone scaffold^27^ and nerve conduit^28^. Chemical gelation of gelatin in the presence of the cross-linker BVSM in a two-step reaction mechanism revealed the ability to control the mechanical properties and degradation rate of gelatin^29^. Several studies showed that cross-linked polymers often affect hydrophilicity and reduce cell adhesion. Wettability plays a key role in determining the biological behavior of a biomaterial, and thus is a critical factor when designing biomaterials^30^. Cellular behaviors were enhanced when cells were grown on specimens with a water contact angle that was lower than 75°^31^. Our data further indicated that gelatin-BVSM materials were hydrophilic^32^. In addition, gelatin-BVSM conduits show consistent microstructures that formed dense and closely-packed walls. This unique property prevents external soft tissues such as scars or connective tissues from invading the internal lumen of the conduits, which can deteriorate or prevent nerve regeneration. Our degradation test showed significant results (P < 0.05). Weight losses of approximately 12.1%, 26.6%, and 76.8% were observed in the 1, 2, and 4 weeks post-implantation specimens, respectively. It is well-known that biomaterials for clinical use must be biodegradable^33,34^. Gelatin-based biomaterials have been shown to enhance mechanical properties and have a controllable degradation rate^35,36^. We hypothesized that a larger surface area and pore volume played a role in the increased degradation of gelatin-BVSM conduits^14^.

Cytotoxic testing and a TUNEL assay were conducted to assess the affinity of gelatin-BVSM to Schwann cells. We found that extracts of this material preserved the growth ability of co-cultured Schwann cells compared to in the control groups. Additionally, gelatin-BVSM is a very compatible biomaterial, as it only invoked a slight inflammatory response. This was observed by both real-time non-invasive NF-κB bioluminescence imaging and histochemical assessment. Thus, gelatin-BVSM is a promising material for peripheral nerve regeneration^28^.

Electromyography is a diagnostic tool designed to investigate the health of muscle and nerve cells by measuring the amplitude and morphology of the electrical signal produced by the skeletal muscles. The electrical signal is measured as a motor unit potential and depends on the density of the muscle fibers attached to that specific motor neuron. Motor unit potentials are recorded during motor conduction studies, which are conducted by stimulating a motor serve and recording the response from the target muscles. During motor conduction studies, the electrical signal (motor unit potential) is generated by the muscle; therefore, it is normal for the results to have high values^37^. In the present study, the electrophysiological data showed that several nerve functions were significantly improved in the gelatin-BVSM group, including NCV and latency, compared to in the silicone rubber conduits. However, the differences in amplitude and MAP area among the entire group comparisons were not significant, indicating that muscle atrophy remained a serious problem even after the muscle fibers had been reinnervated.

CGRP is a neuropeptide found in both the central and peripheral nervous systems. CGRP is primarily synthesized in DRG cell bodies and transported axonally to the endings of nerve fibers in the central and peripheral nervous systems^38^. Previous studies reported that CGRP is a peptide related to nerve regeneration whose functions include suppressing specific immune cells such as T-lymphocytes. Numerous studies have characterized the stimulatory effects of CGRPs on neurons as well as its effects on the survivability rate of injured neurons and rate of neuronal loss^39^. BDNF plays an important role in modulating neuroplasticity and promoting better and efficient recovery after spinal cord injuries^40^. Several studies have reported the potency of BNDF and IGF in maintaining and preventing hypoxic-ischemic-induced brain damage to neuronal cells^41^. Immediately after post-nerve injuries, the expression of neurotrophic factors such as GDNF increases. Simultaneously, there is a period in which the levels of neurotrophic factors receptors in Schwann cells and neurons are increased before returning to normal values. In addition, regional and constant application of exogenous GDNF greatly promotes local axon regeneration and recovery after delayed nerve repair^42^. In the present study, the dorsal horn on the similar side of injury showed the highest expression of CGRP at 5 weeks post-implantation, which subsequently decreased from weeks 5 to 8. The decreasing CGRP expression at week 8 may have occurred because regenerated nerves were in the final stage of regeneration, and thus were more mature, stimulating less injury-related proteins to the neurons; similar results were observed for the silicone rubber conduits. Morphometric studies also supported that the microstructure of regenerated nerves became more mature with increasing myelinated axons in larger nerve areas over postoperative periods in the bridging conduits.

Successful PNS regeneration relies on multiple cell types, including damaged axons and non-neuronal cells such as immune cells, particularly macrophages. Upon nerve injury, macrophages that infiltrate injury sites not only contribute to Wallerian degeneration, but also are polarized to different phenotypes to affect axonal regeneration. In the present study, gelatin-BVSM conduits were found to induce lesser macrophage infiltration into the regenerated nerves during the inflammatory period. This revealed that the gelatin-BVSM conduits has stable properties and does not induce serious inflammatory reactions, thus providing a consistent and satisfactory support for growing axons as observed for silicone rubber conduits.

This is the first study to examine the use of gelatin-BVSM used for newly nerve conduits. The experimental test details, used in several recent studies on biodegradable bridging conduits to repair injured rat sciatic nerves, were gleaned from the literature and summarized (Table 2). Our results demonstrate that gelatin-BVSM has strong mechanical properties, biodegradation, and low toxicity, and shows potential for applications such as nerve-bridging conduits. *In vivo* data showed that gelatin-BVSM recruited moderate macrophage infiltration, expression of CGRP, and neuron-related growth factors, such as IGF-1, BDNF, and GDNF for nerve regeneration. These findings suggest that this novel gelatin-BVSM nerve conduit is suitable for treatment of injured peripheral nerve defects.

**Table 2:** Experimental details of recent studies of biodegradable bridging conduits to repair injured rat sciatic nerves.

Materials	Crosslinking agent	Gap (mm)	Myelinated axons	Findings	Ref
Gelatin	EDC	5	GAP-43 showed high expression in conduit group.	Porous conduits improved regeneration of the injured nerve.	^43^
Chitosan	EDC/genipin	10	The distal nerve showed greater myelinated axons regeneration after 6 weeks	The chitosan conduit promoted regeneration of peripheral nerve fibers.	^44^
PCL	EDC/NHS	10	Axonal extension across the entire length was expected after 8 weeks.	The results of tissue regeneration were similar to those of the autograft.	^45^
Collagen	EDC	10	Most nerves were matured and densely arranged with a uniform structure after 20 weeks.	These results indicate that the hybrid conduit can serve as a new artificial graft.	^46^
Elastin	Glutaraldehyde	10	The nerves were matured after 10 weeks in the conduit groups.	The study suggests that functional elastin can be used as a biomaterial for peripheral nerve regeneration.	^47^
Gelatin	Glutaraldehyde	10	The nerves were matured after 3 months in the conduit groups.	The scaffold featured a partially fenestrated outer layer, although it remained circular. The regenerated nerves became more mature.	^48^
